# Supplementary material for: MYC competes with MiT/TFE in regulating lysosomal biogenesis and autophagy through an epigenetic rheostat
Source: Nat Commun. 2019 Aug 9;10:3623. doi: 10.1038/s41467-019-11568-0 (PMC6689058; doi:10.1038/s41467-019-11568-0)
Supplement: Supplementary file 1 — Supplementary Information [file 41467_2019_11568_MOESM1_ESM.pdf]

MYC competes with MiT/TFE in regulating lysosomal biogenesis and autophagy through an epigenetic rheostat

Supplementary Information

Annunziata et al.

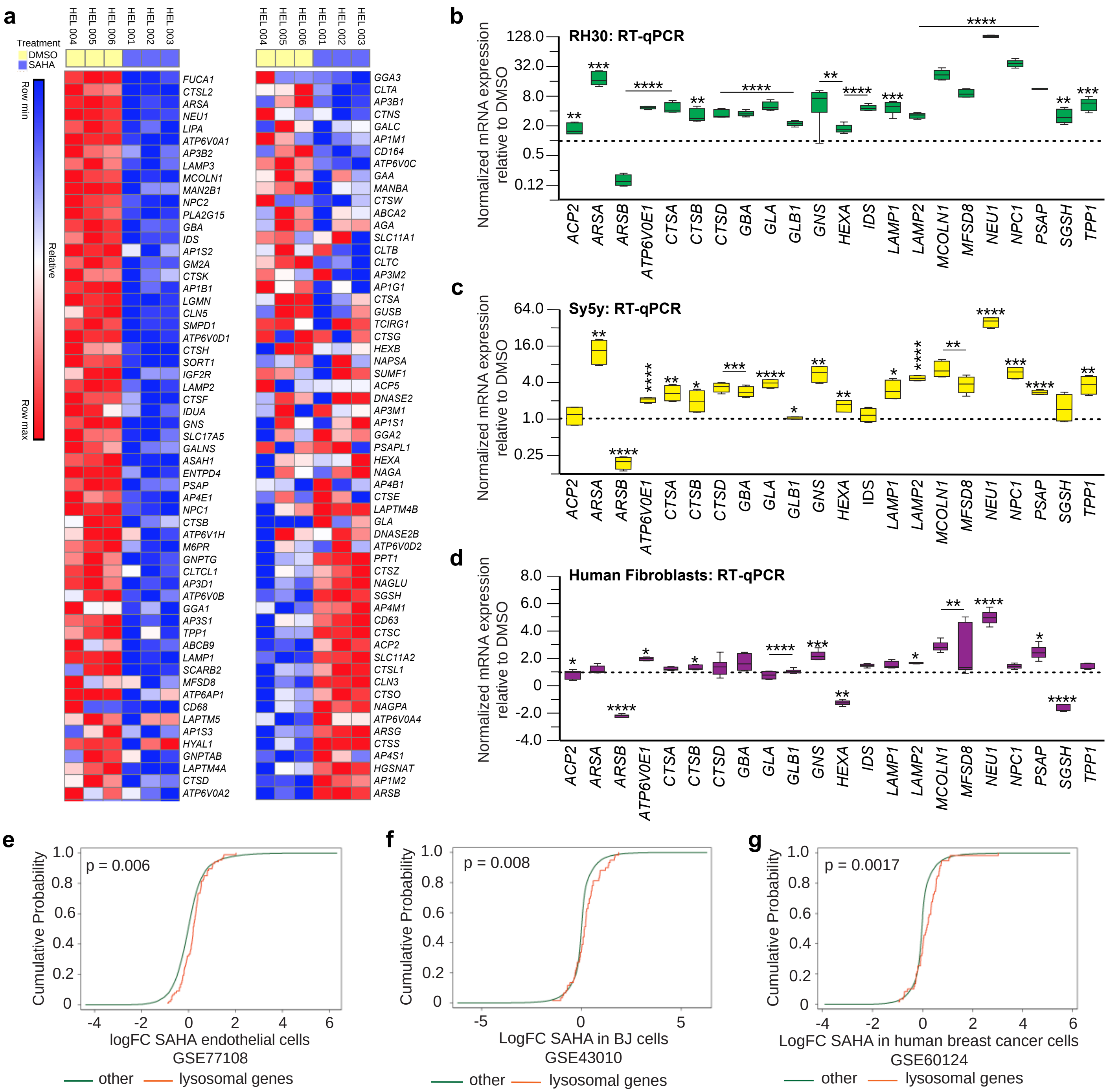

Supplementary Figure 1. Lysosomal genes are regulated by HDAC inhibition. **a** Heat map of genes within the KEGG lysosome pathway was obtained from microarray analysis of HeLa cells treated with SAHA (20μM for 24h) or DMSO. **b, c, d** Expression analyses of lysosomal genes in

rhabdomyosarcoma cells (**b**) (RH30; n=5); in neuroblastoma cells (**c**) (Sy5y, n=4); in skin fibroblasts from control individuals (**d**) (n=9) upon SAHA treatment (20μM for 24h). The Box and whisker plots are shown as the normalized expression of the mRNA of lysosomal genes in SAHA-treated cells relative to that in DMSO-treated cells. Boxes represent the mean value and bar inside the box represents median value; upper bar represents maximum of distribution, lower bar represents minimum of distribution (95% confidence level). **e-g** Lysosomal gene expression obtained from **e** (GSE77108) endothelial-, **f** (GSE43010) transformed lymphoblastoid-, and **g** (GSE60124) breast cancer-cells treated with SAHA. In GSE77108: SAHA 2 μM for 12 hours; in GSE43010: SAHA 50 *μ*M for 72 hours; in GSE60124: SAHA 7.5 μM for 9 hours.

Graphs in **b-d** are presented as the mean ± SD. Statistical analyses were performed using the Student *t*-test. *p<0.05, **p<0.01, ***p<0.001, ****p<0.0001.

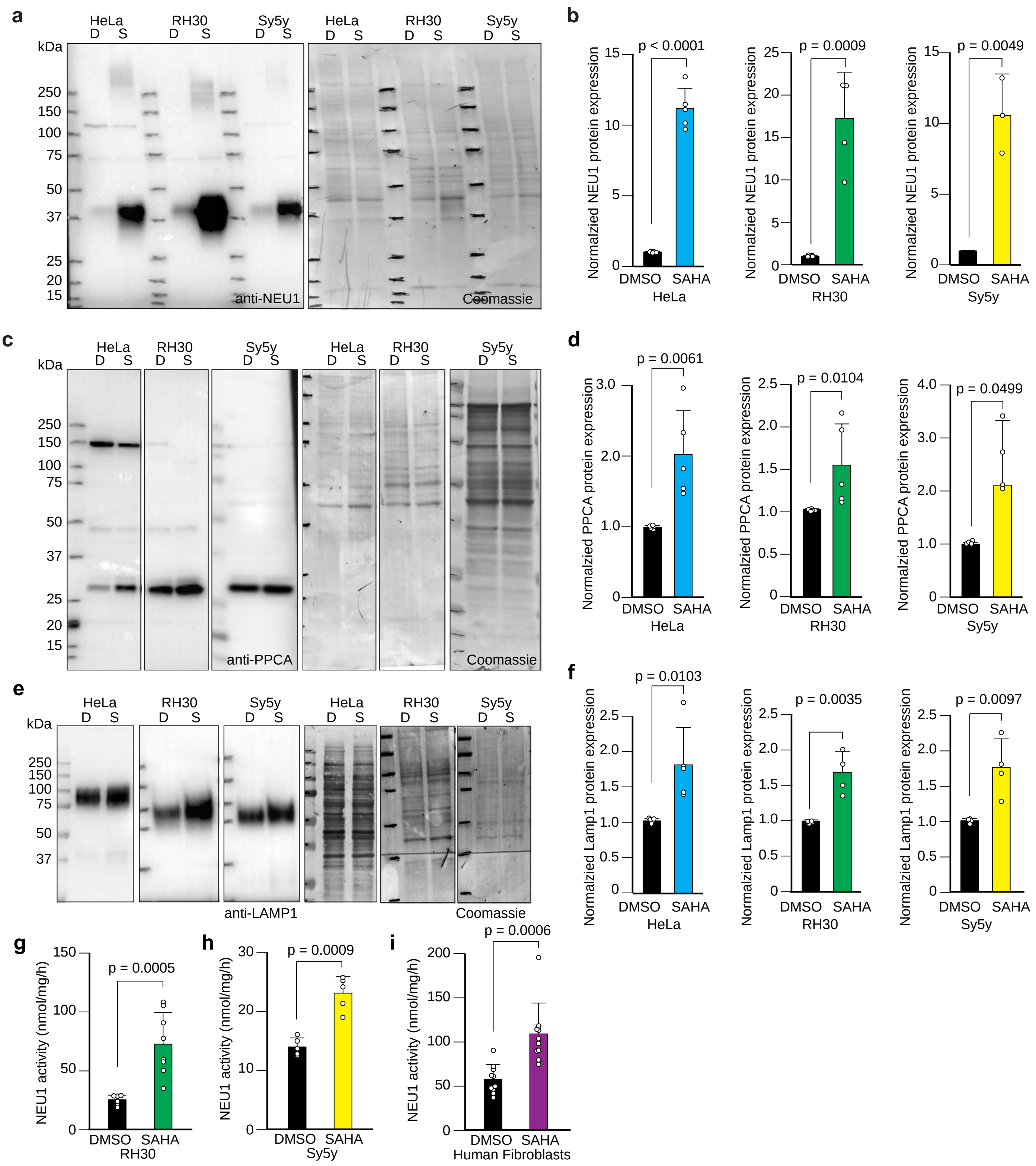

a
HeLa D S
RH30 D S
Sy5y D S
kDa
250
150
100
75
50
37
25
20
15
anti-NEU1
Coomassie
b
p < 0.0001
p = 0.0009
p = 0.0049
Normalzied NEU1 protein expression
DMSO SAHA
HeLa
RH30
Sy5y
c
anti-PPCA
Coomassie
d
p = 0.0061
p = 0.0104
p = 0.0499
Normalzied PPCA protein expression
e
anti-LAMP1
Coomassie
f
p = 0.0103
p = 0.0035
p = 0.0097
Normalzied Lamp1 protein expression
g
p = 0.0005
NEU1 activity (nmol/mg/h)
RH30
h
p = 0.0009
Sy5y
i
p = 0.0006
Human Fibroblasts

Supplementary Figure 2. HDAC inhibition induces lysosomal biogenesis. **a** Left, representative immunoblots from HeLa, RH30 and Sy5y cells treated with DMSO (D) or SAHA (S) (20μM for 24h) probed with anti-NEU1 antibody; right, Coomassie stained blot used as loading control. **b** Quantification of NEU1 levels in HeLa (n=5), RH30 (n=4) and Sy5y (n=3) normalized to loading control. **c** Left, representative immunoblots from HeLa, RH30 and Sy5y cells treated with DMSO (D) or SAHA (S) (20μM for 24h) probed with anti-PPCA antibody. Right, Coomassie stained blot was used as loading control. **d** Quantification of PPCA levels in HeLa (n=5), RH30 (n=5) and Sy5y (n=4) normalized to loading control. **e** Left, representative immunoblots from HeLa, RH30 and Sy5y cells treated with DMSO (D) or SAHA (S) (20μM for 24h) probed with anti-LAMP1 antibody. Right, Coomassie stained blot was used as loading control. **f** Quantification of LAMP1 levels in HeLa (n=5), RH30 (n=4) and Sy5y (n=4) normalized to loading control. **g,h,i** NEU1 activity was measured in **g** RH30 (n=8), **h** Sy5y (n=5) and **i** human fibroblasts (n=10) treated with DMSO or SAHA (20μM for 24h).

Graphs are presented as the mean ± SD. Significance was evaluated with Student's *t*-test.

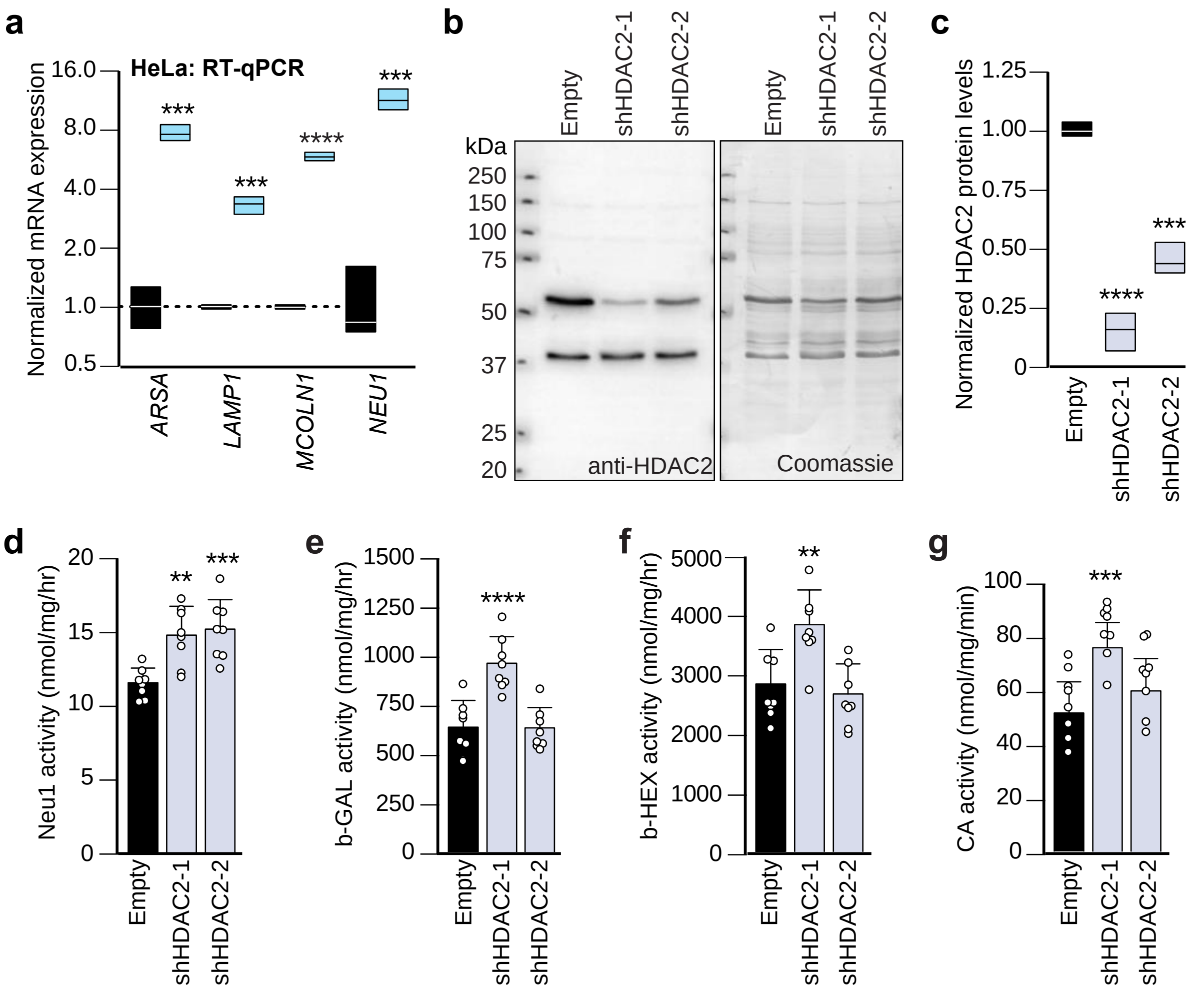

Supplementary Figure 3. HDAC2 silencing enhances the activity of lysosomal enzymes. **a** Expression of several lysosomal genes upon treatment of HeLa with romidepsin (10nM for 24h). The box and whisker plot show normalized expression of the lysosomal mRNAs in romidepsin-treated HeLa cells and in DMSO-treated cells; (n=3). **b** Left, silencing of HDAC2 in HeLa cells with two shRNA preparations (shHDAC2-1, shHDAC2-2). Right, Coomassie stained immunoblot probed with anti-HDAC2 was used as loading control. **c** Quantification of HDAC2 levels in HDAC2-silenced HeLa cells normalized to loading control (n=3). **d-g** Activity assays for **d** NEU1, **e** β-galactosidase (β-GAL), **f** β-hexosaminidase (β-HEX), **g** cathepsin A (CA) in HDAC2-silenced HeLa cells (n=8).

Boxes represent the mean value and bar inside the box represents median value; upper bar represents maximum of distribution, lower bar represents minimum of distribution (95% confidence level). Graphs are presented as mean ± SD. Statistical analyses were performed using the Student *t*-test. *$p<0.05$, **$p<0.01$, ***$p<0.001$, ****$p<0.0001$.

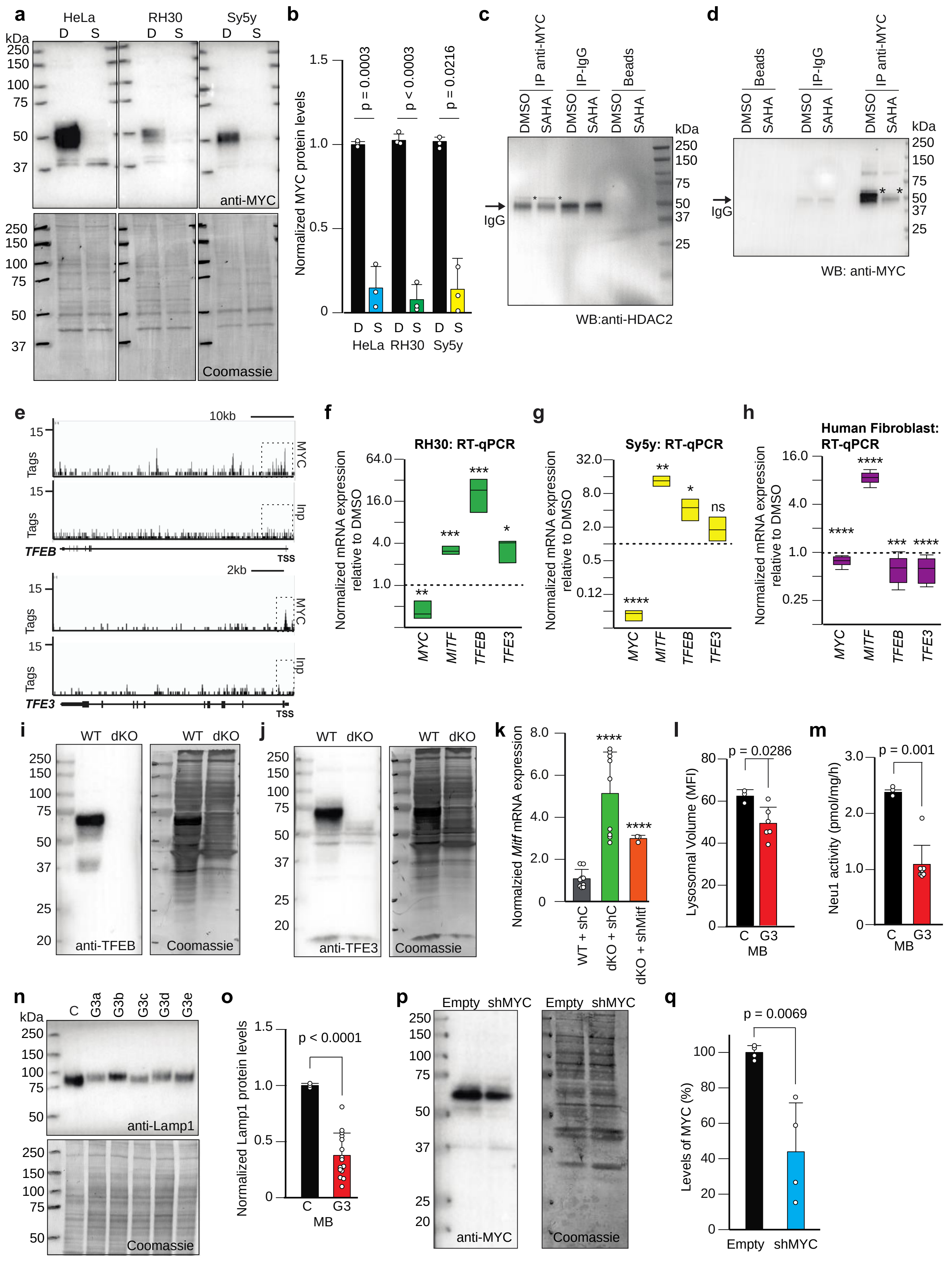

a
HeLa
RH30
Sy5y
D S
kDa
250
150
100
75
50
37
anti-MYC
Coomassie
b
Normalized MYC protein levels
p = 0.0003
p < 0.0003
p = 0.0216
D S D S D S
HeLa RH30 Sy5y
c
IP anti-MYC
IP-IgG
Beads
DMSO
SAHA
IgG
WB:anti-HDAC2
d
WB: anti-MYC
e
10kb
Tags
MYC
Inp
TFEB
TSS
2kb
TFE3
f
RH30: RT-qPCR
Normalized mRNA expression relative to DMSO
MYC
MITF
TFEB
TFE3
g
Sy5y: RT-qPCR
h
Human Fibroblast: RT-qPCR
i
WT dKO
anti-TFEB
Coomassie
j
anti-TFE3
k
Normalized Mitf mRNA expression
WT + shC
dKO + shC
dKO + shMitf
l
Lysosomal Volume (MFI)
p = 0.0286
C G3
MB
m
Neu1 activity (pmol/mg/h)
p = 0.001
n
C G3a G3b G3c G3d G3e
anti-Lamp1
o
Normalized Lamp1 protein levels
p < 0.0001
p
Empty shMYC
anti-MYC
q
p = 0.0069
Levels of MYC (%)
Empty shMYC

Supplementary Figure 4. HDAC inhibition regulates MYC levels and the expression of the MiT/TFE transcription factors. **a** Top, MYC levels in SAHA-treated HeLa, RH30, and Sy5y (20μM/24h). Bottom, Coomassie staining used as loading control. D=DMSO, S=SAHA. **b** Quantification of MYC levels in SAHA-treated HeLa, RH30, and Sy5y (20μM/24h; n=3) normalized to loading control. **c,d** Full blots of co-immunoprecipitated MYC and HDAC2 shown in Fig. 2d,e. **e** MYC binding to *TFEB* and *TFE3* promoters in U2OS cells overexpressing MYC. Peaks are marked by dotted rectangles; Input DNA (Inp) serves as reference. **f-h** Expression of *MYC* and *MIT/TFE* after SAHA treatment (20μM/24h) in **f** RH30 (n=3), **g** Sy5y (n=3) and **h** normal fibroblasts (n=9). **i,j** Immunoblots of lysates from MEFs WT or Tfeb and Tfe3 dKO probed with **i** anti-TFEB and **j** anti-TFE3 antibodies. Right, Coomassie-stained membranes used as loading control. **k** Expression of Mitf in MEFs WT (n=9), dKO (n=9) and dKO with silenced Mitf (n=3). shC refers to cells transduced with shRNA control lentivirus. **l** Lysosomal volume measured by FACS analysis as mean fluorescence intensity (MFI) after staining with Lysotracker medulloblastoma tumorspheres *Trp53*$^{-/-}$ overexpressing *Myc*) (n=6) or *Trp53*$^{-/-}$ controls (n=3). **m** Neu1 activity in tumorspheres (n=6) and in controls (n=3). **n** Top, Immunoblot of Lamp1 in tumorspheres (G3a-G3e) and in control. Bottom, Coomassie-stained membrane used as loading control. **o** Quantification of Lamp1 levels in tumorspheres (n=15) and in controls (n=3). **p** Left, Immunoblot of HeLa cells silenced for *MYC* (shMYC). Right, Coomassie-stained membrane used as loading control. **q** Quantification of MYC levels in MYC-silenced HeLa cells normalized to loading control (n=4).

Boxes represent the mean value and bar inside the box represents median value; upper bar represents maximum of distribution, lower bar represents minimum of distribution (95% confidence level). Graphs are presented as mean ± SD. Statistical analyses were performed using the Student *t*-test. *p<0.05, **p<0.01, ***p<0.001, ****p<0.0001.

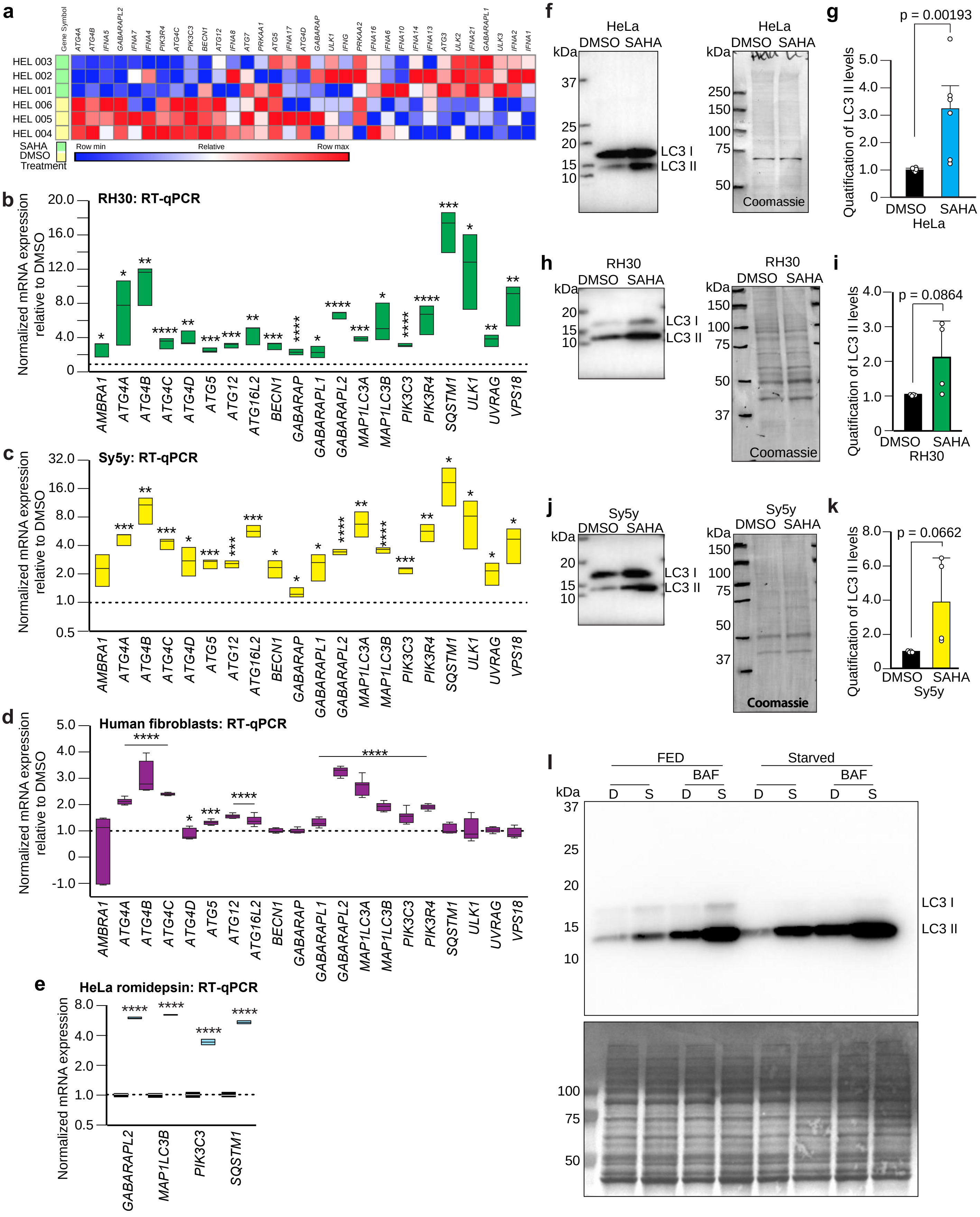
a
Gene Symbol
ATG4A ATG4B IFNA5 GABARAPL2 IFNA7 IFNA4 PIK3R4 ATG4C PIK3C3 BECN1 ATG12 IFNA8 ATG7 PRKAA1 ATG5 IFNA17 ATG4D GABARAP ULK1 IFNG PRKAA2 IFNA16 IFNA6 IFNA10 IFNA14 IFNA13 ATG3 ULK2 IFNA21 GABARAPL1 ULK3 IFNA2 IFNA1
HEL 003
HEL 002
HEL 001
HEL 006
HEL 005
HEL 004
SAHA
DMSO
Treatment
Row min
Relative
Row max
b
RH30: RT-qPCR
Normalized mRNA expression relative to DMSO
20.0
16.0
12.0
8.0
4.0
0
AMBRA1 ATG4A ATG4B ATG4C ATG4D ATG5 ATG12 ATG16L2 BECN1 GABARAP GABARAPL1 GABARAPL2 MAP1LC3A MAP1LC3B PIK3C3 PIK3R4 SQSTM1 ULK1 UVRAG VPS18
c
Sy5y: RT-qPCR
Normalized mRNA expression relative to DMSO
32.0
16.0
8.0
4.0
2.0
1.0
0.5
AMBRA1 ATG4A ATG4B ATG4C ATG4D ATG5 ATG12 ATG16L2 BECN1 GABARAP GABARAPL1 GABARAPL2 MAP1LC3A MAP1LC3B PIK3C3 PIK3R4 SQSTM1 ULK1 UVRAG VPS18
d
Human fibroblasts: RT-qPCR
Normalized mRNA expression relative to DMSO
5.0
4.0
3.0
2.0
1.0
0
-1.0
AMBRA1 ATG4A ATG4B ATG4C ATG4D ATG5 ATG12 ATG16L2 BECN1 GABARAP GABARAPL1 GABARAPL2 MAP1LC3A MAP1LC3B PIK3C3 PIK3R4 SQSTM1 ULK1 UVRAG VPS18
e
HeLa romidepsin: RT-qPCR
Normalized mRNA expression
8.0
4.0
2.0
1.0
0.5
GABARAPL2 MAP1LC3B PIK3C3 SQSTM1
f
HeLa
DMSO SAHA
kDa
37
25
20
15
10
LC3 I
LC3 II
HeLa
DMSO SAHA
kDa
250
150
100
75
50
Coomassie
g
p = 0.00193
Quatification of LC3 II levels
5.0
4.0
3.0
2.0
1.0
0
DMSO SAHA
HeLa
h
RH30
DMSO SAHA
kDa
20
15
10
LC3 I
LC3 II
RH30
DMSO SAHA
kDa
150
100
75
50
37
Coomassie
i
p = 0.0864
Quatification of LC3 II levels
4.0
3.0
2.0
1.0
0
DMSO SAHA
RH30
j
Sy5y
DMSO SAHA
kDa
20
15
10
LC3 I
LC3 II
Sy5y
DMSO SAHA
kDa
150
100
75
50
37
Coomassie
k
p = 0.0662
Quatification of LC3 II levels
8.0
6.0
4.0
2.0
0
DMSO SAHA
Sy5y
l
FED
Starved
BAF
BAF
kDa
D S D S D S D S
37
25
20
15
10
LC3 I
LC3 II
100
75
50

Supplementary Figure 5. SAHA treatment induces autophagy. **a** Heat map of KEGG autophagy genes in HeLa cells upon DMSO and SAHA treatment (20μM for 24h). **b-d** Expression analyses of autophagy genes upon SAHA treatment (20μM for 24h) of **b** RH30 (n=3); **c** Sy5y (n=3); and **d** skin fibroblasts from control individuals (n=9). **e** Expression of several autophagy genes upon treatment of HeLa with romidepsin (10nM for 24h) (n=3). The box and whisker plot are shown as the normalized expression of the lysosomal mRNAs in SAHA- or Romidepsin-treated HeLa cells and in DMSO-treated cells. Boxes represent the mean value and bar inside the box represents median value; upper bar represents maximum of distribution, lower bar represents minimum of distribution (95% confidence level). **f** Left, levels of LC3 protein in HeLa. Right, Coomassie staining used as loading control. **g** Quantification of LC3II/LC3I ratio in HeLa normalized to loading control (n=6). **h** Left, representative immunoblot of RH30 probed with anti LC3 antibody upon treatment with SAHA (20μM for 24h); right, Coomassie staining used as loading control. **i** Quantification of LC3II/LC3I ratio in RH30 normalized to loading control (n=4). **j** Left, representative immunoblot of Sy5y probed with anti-LC3 antibody upon treatment with SAHA (20μM for 24h); right, Coomassie staining used as loading control. **k** Quantification LC3II/LC3I ratio in Sy5y normalized to loading control (n=4). **l** Full length immunoblot probed with anti-LC3 (top) and Coomassie-stained membrane (bottom) shown cropped in Fig. 5d.

Graphs are presented as mean ± SD. Statistical analyses were performed using the Student *t*-test. p<0.05, **p<0.01, ***p<0.001, ****p<0.0001.

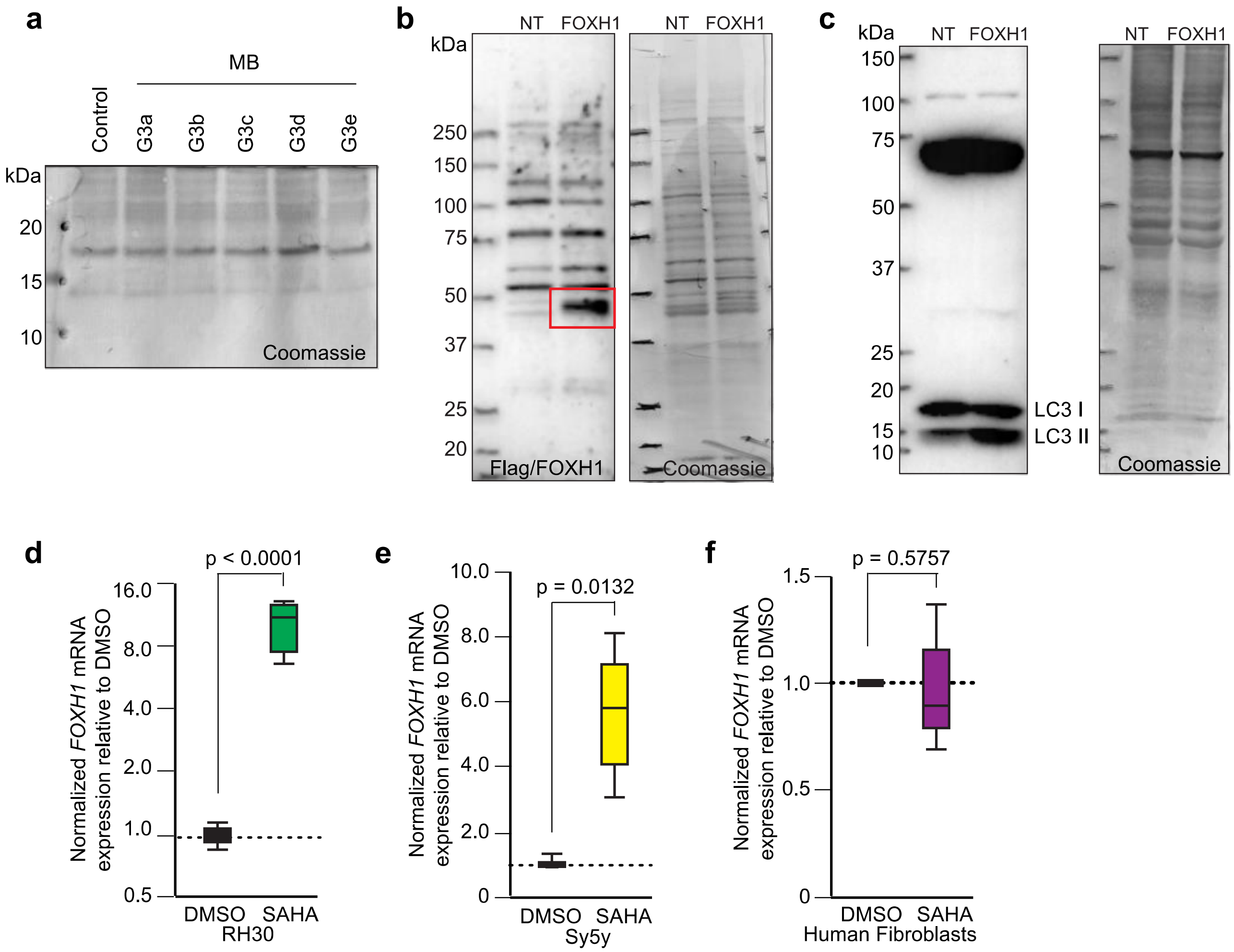

Supplementary Figure 6. Autophagy markers are induced by romidepsin treatment and by FOXH1. **a** Coomassie staining of blot shown in Fig.6d used as loading control (n=6). **b** Left, the levels of Flag-tagged FOXH1 were evaluated in HeLa cells transiently transfected with FOXH1 and compared to non- transfected cells (NT), right Coomassie staining used as control. **c** full blot and Coomassie staining of corresponding membrane shown as partial in Fig. 7c. **d-f** Expression analyses of *FOXH1* after SAHA treatment (20μM for 24h) in **d** RH30 (n= 5); **e** Sy5y (n=3); **f** skin fibroblasts from controls (n=10). The Box and Whisker plots are shown as the expression of the mRNA of FOXH1 in SAHA-treated cells relative to that of DMSO-treated cells. Boxes represent

the mean value and bar inside the box represents median value; upper bar represents maximum of distribution, lower bar represents minimum of distribution (95% confidence level).

Graphs are presented as mean ± SD. Statistical analyses were performed using the Student *t*-test.

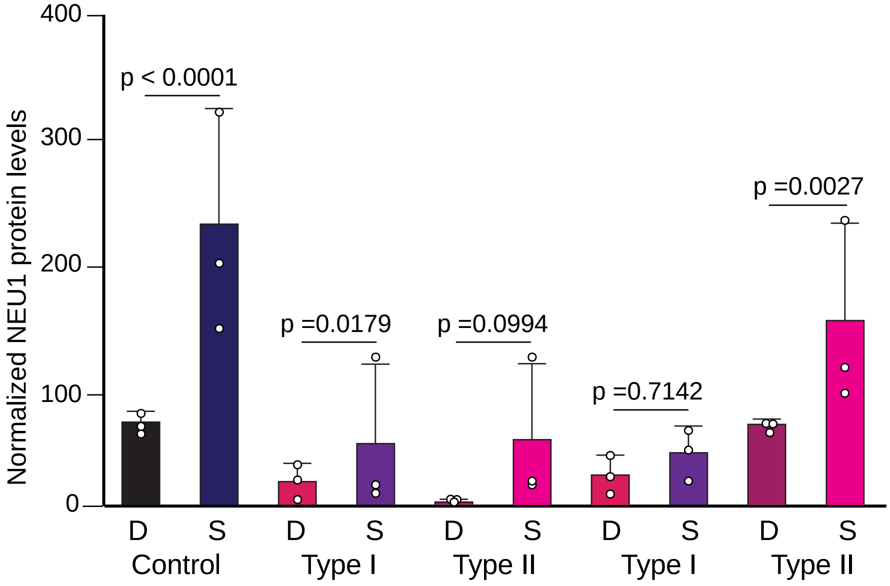

Supplementary Figure 7. NEU1 levels in sialidosis fibroblasts increase upon SAHA treatment. Quantification of NEU1 protein levels after SAHA treatment (SAHA, 20μM for 24h) in sialidosis fibroblasts normalized to loading control shown in Fig. 8 (n=3). Type I = attenuated form of sialidosis; Type II = severe form of sialidosis.

Graphs are presented as mean ± SD. Statistical analyses were performed using the Student *t*-test.

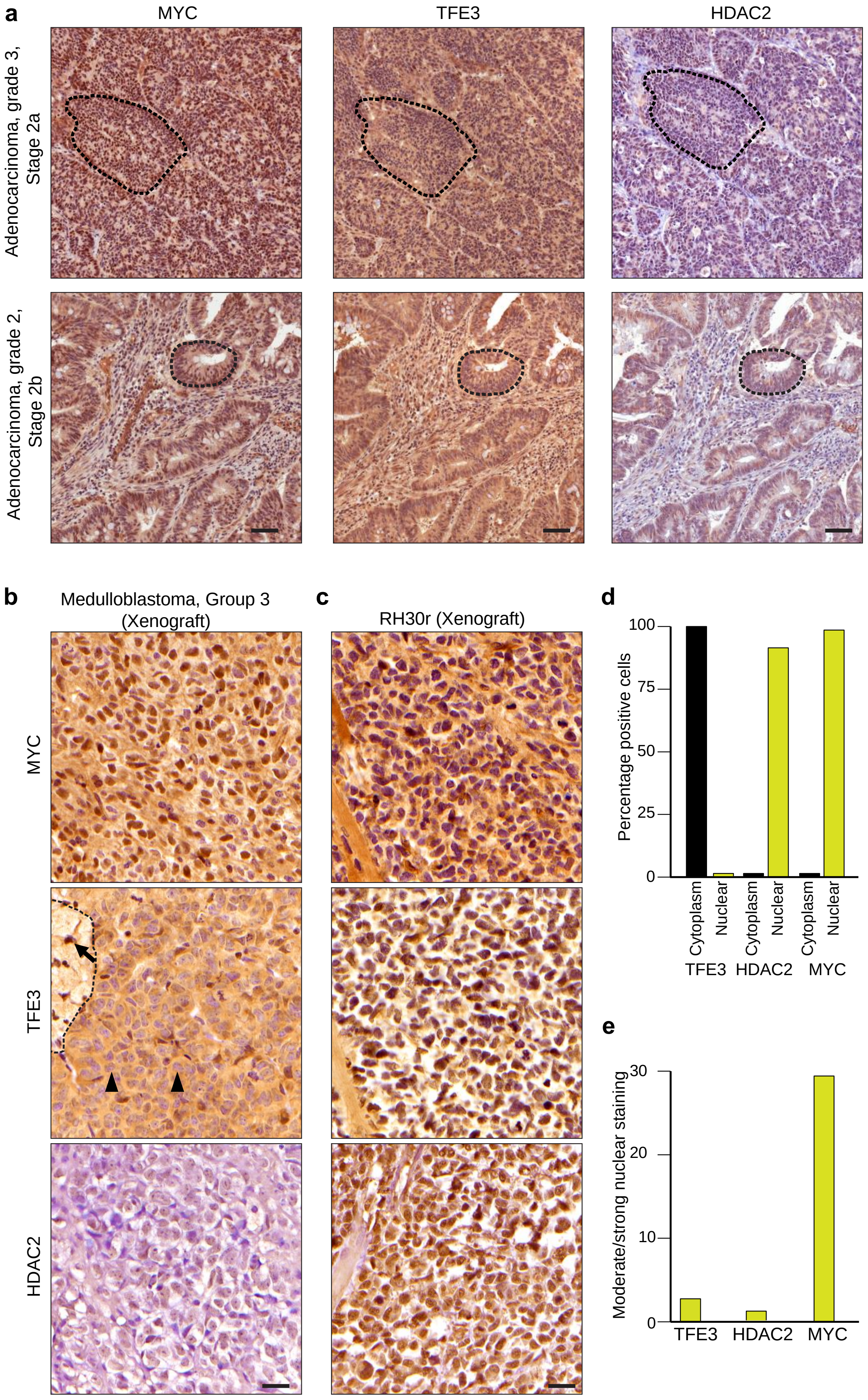
a
MYC
TFE3
HDAC2
Adenocarcinoma, grade 3, Stage 2a
Adenocarcinoma, grade 2, Stage 2b
b
Medulloblastoma, Group 3 (Xenograft)
c
RH30r (Xenograft)
MYC
TFE3
HDAC2
d
Percentage positive cells
100
75
50
25
0
Cytoplasm
Nuclear
Cytoplasm
Nuclear
Cytoplasm
Nuclear
TFE3
HDAC2
MYC
e
Moderate/strong nuclear staining
30
20
10
0
TFE3
HDAC2
MYC

Supplementary Figure 8. MYC antagonizes MiT/TFE in cancer**. a** Expression of MYC, TFE3 and HDAC2 in colon adenocarcinoma samples. Representative immunohistochemistry images of two colon cancer samples labeled for MYC, TFE3 or HDAC2. Outlined areas highlight subpopulations of neoplastic cells with MYC, HDAC2 nuclear expression and TFE3 cytoplasmic distribution. Scale bar 50 μm. **b** Expression of MYC, TFE3 and HDAC2 in a group 3 medulloblastoma patient-derived xenograft. The dashed line in the TFE3 image separates engrafted neoplastic cells from a focus of histiocytic inflammation. Neoplastic cells lack nuclear expression of TFE3 (black arrowheads) as assayed by IHC while histiocytic infiltrates have positive nuclear labeling (black arrows), which serves as one internal control for assessing TFE3 labeling and its subcellular localization. Scale bar 25 μm magnification. **c** Expression of MYC, TFE3 and HDAC2 in the rhabdomyosarcoma patient-derived xenograft RH30r. Scale bar 25 μm. **d** Expression of MYC, TFE3 and HDAC2 in the cytoplasm and the nucleus of the group 3 medulloblastoma patient-derived xenograft shown in b. **e** Nuclear staining of MYC, TFE3 and HDAC2 in the RH30r xenograft shown in c.

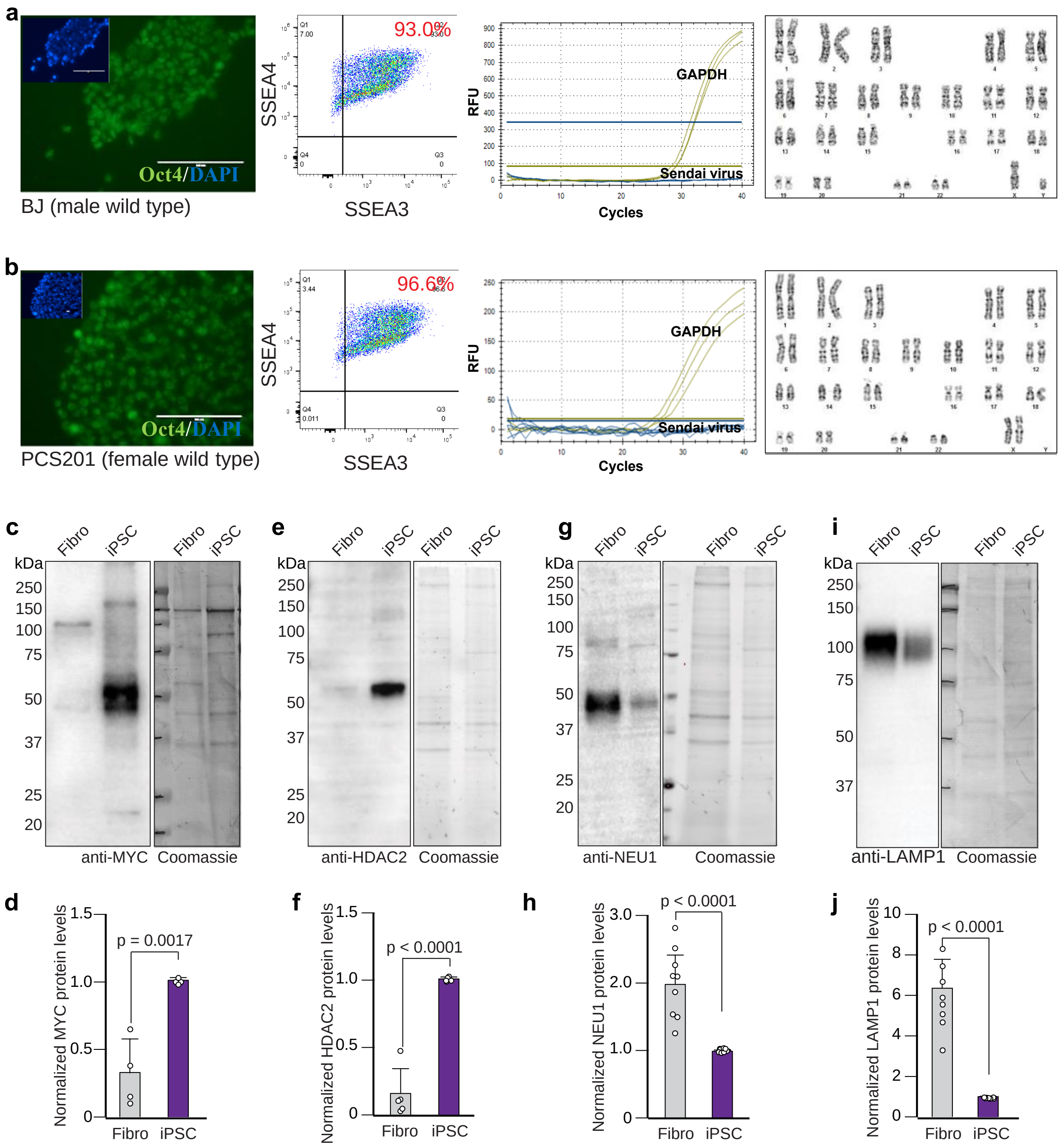

Supplementary Figure 9. Characterization of pluripotent stem cells. **a,b** generation of hiPSCs from human fibroblasts with the Sendai virus technology of BJ (male wild type) and PCS201 (female wild type), respectively. Pluripotency of hiPSCs was confirmed with immunofluorescence

assays performed with anti-Oct4 (green) antibody (left). DAPI (blue) was used to visualize nuclei. FACS analysis of SSEA3/SSEA4 defined pluripotency of hiPSCs (center). No obvious genomic integration was found by qPCR with Sendai virus specific primers (third panel from the left). G banded chromosome analysis showing normal karyotype (right). **c,e,g,i** Full immunoblots of hiPSCs and their parental fibroblasts probed with **c** anti-MYC, **e** anti-HDAC2, **g** anti-NEU1 and **i** anti-LAMP1 antibodies, shown in Fig.9f. **d,f,h,j** Quantification of the levels of **d** MYC (n=4), **f** HDAC2 (n=5), **h** NEU1 (n=9) and **j** LAMP1 (n=8) in hiPSCs and their parental fibroblasts normalized to loading control.

Values are presented in graph as the mean ± SD; statistical analyses were performed using the Student *t*-test.

SUPPLEMENTARY TABLES

Supplementary Table 1. Top Pathway Enriched among Upregulated Genes per DAVID Enrichment Analysis

Category	Term	Count	%	p-Value	Genes	Fold Enrichment	Benjamini
KEGG_ PATHWAY	hsa04142: Lysosome	32	1.59	5.30E-07	CTSL2, GM2A, AP1B1, AP4E1, LGMN, ASAH1, IDS, GALNS, AP3B2, AP3D1, ENTPD4, MAN2B1, ATP6V0D1, GBA, PLA2G15, LIPA, PSAP, FUCA1, M6PR, GNS, LAMP2, SLC17A5, NPC1, LAMP3, ARSA, SMPD1, SORT1, ATP6V0A1, NEU1, CTSH, CLN5, CTSF	2.62	9.59E-05

Supplementary Table 2. MYC Binding to Lysosomal Genes

		Genes	Lysosomes	Total
Not bound	0	13,212	33	13,245
Bound	1	13,284	80	13,364
	Total	26,496	113	26,609

Fisher’s exact = 0.0001, 1-sided Fisher’s exact = 0.0001, di r(p_exact) 0.00001124. Data obtained from [29]

Supplementary Table 3. MYC Binding to MiT/TFE Transcription Factors

Chr	Start	End	Tss	Bound[a]	Symbol
Chr3	69788353	69788711	69788585	1	MITF
Chr3	69788353	69788711	69812706	0	MITF
Chr3	69788353	69788711	69812961	0	MITF
Chr3	69788353	69788711	69915374	0	MITF
Chr3	69788353	69788711	69985750	0	MITF
Chr6	48900685	48900936	48900990	1	TFEB
ChrX	41702429	41702659	41702798	1	TFE3

[a]Bound = 1, Not bound = 0, Data obtained from [29] .

Supplementary Table 4. Correlation Analysis for FOXH1 Binding to a Subset of Autophagy Genes Using the Microarray Dataset from GSE50206

**Column ID**	**r**	**p-value (correlation to FOXH1)**
SIRT1	0.788636	6.2029e-015
FKBP1B	0.75354	4.38367e-013
ATG16L2	0.635446	1.29759e-008
MAPK1	0.625874	2.47469e-008
USP10	0.610577	6.64345e-008
MTMR14	0.561582	1.13349e-006
ATG10	0.455157	0.00013929
ATG4C	0.389292	0.00135004
ATG5	0.353234	0.00389826
UVRAG	0.275	0.0266205
