# Supplementary material for: MYC competes with MiT/TFE in regulating lysosomal biogenesis and autophagy through an epigenetic rheostat
Source: Nat Commun. 2019 Aug 9;10:3623. doi: 10.1038/s41467-019-11568-0 (PMC6689058; doi:10.1038/s41467-019-11568-0)
Supplement: Supplementary file 3 — Description of Additional Supplementary Files [file 41467_2019_11568_MOESM3_ESM.pdf]

Description of Additional Supplementary Files

File Name: Supplementary Data 1
Description: **Top Upregulated and Downregulated Pathways in SAHA-Treated Cells (GSEA)**

File Name: Supplementary Data 2
Description: **Analysis of MYC Binding to Lysosomal Genes in MYC-Overexpressing Medulloblastoma Cells**. a Myc binding measured by ChIP-seq; the value indicates binding strength at the promoter. NB = not bound, NA = not annotated; b Regulation after activating MYC-ER by tamoxifen. Value is a log2-fold change. Mean of the 12-h and 24-h timepoints was compared to that of the 0-h time point. NA = not annotated; Data obtained from [29]

File Name: Supplementary Data 3
Description: **Analysis of MYC Binding to Autophagy Genes in MYC-Overexpressing Medulloblastoma Cells**. a Myc binding measured by ChIP-seq; the value indicates binding strength at the promoter. NB = not bound; b Regulation after activating MYC-ER by tamoxifen. Value is a log2-fold change. Mean of the 12-h and 24-h timepoints was compared with that of the 0-h timepoint. Data obtained from [29]
