# Supplementary material for: MYC competes with MiT/TFE in regulating lysosomal biogenesis and autophagy through an epigenetic rheostat
Source: Nat Commun. 2019 Aug 9;10:3623. doi: 10.1038/s41467-019-11568-0 (PMC6689058; doi:10.1038/s41467-019-11568-0)
Supplement: Supplementary file 7 — Reporting Summary [file 41467_2019_11568_MOESM7_ESM.pdf]

nature research

Corresponding author(s): Alessandra d'Azzo

Last updated by author(s): Jun 24, 2019

Reporting Summary

Nature Research wishes to improve the reproducibility of the work that we publish. This form provides structure for consistency and transparency in reporting. For further information on Nature Research policies, see Authors & Referees and the Editorial Policy Checklist.

Statistics

For all statistical analyses, confirm that the following items are present in the figure legend, table legend, main text, or Methods section.

n/a	Confirmed	
☐	☒	The exact sample size (*n*) for each experimental group/condition, given as a discrete number and unit of measurement
☐	☒	A statement on whether measurements were taken from distinct samples or whether the same sample was measured repeatedly
☐	☒	The statistical test(s) used AND whether they are one- or two-sided<br>*Only common tests should be described solely by name; describe more complex techniques in the Methods section.*
☒	☐	A description of all covariates tested
☒	☐	A description of any assumptions or corrections, such as tests of normality and adjustment for multiple comparisons
☐	☒	A full description of the statistical parameters including central tendency (e.g. means) or other basic estimates (e.g. regression coefficient) AND variation (e.g. standard deviation) or associated estimates of uncertainty (e.g. confidence intervals)
☒	☐	For null hypothesis testing, the test statistic (e.g. *F*, *t*, *r*) with confidence intervals, effect sizes, degrees of freedom and *P* value noted<br>*Give P values as exact values whenever suitable.*
☒	☐	For Bayesian analysis, information on the choice of priors and Markov chain Monte Carlo settings
☒	☐	For hierarchical and complex designs, identification of the appropriate level for tests and full reporting of outcomes
☒	☐	Estimates of effect sizes (e.g. Cohen's *d*, Pearson's *r*), indicating how they were calculated

Our web collection on statistics for biologists contains articles on many of the points above.

Software and code

Policy information about availability of computer code

Data collection: *Provide a description of all commercial, open source and custom code used to collect the data in this study, specifying the version used OR state that no software was used.*

Data analysis: *Provide a description of all commercial, open source and custom code used to analyse the data in this study, specifying the version used OR state that no software was used.*

For manuscripts utilizing custom algorithms or software that are central to the research but not yet described in published literature, software must be made available to editors/reviewers. We strongly encourage code deposition in a community repository (e.g. GitHub). See the Nature Research guidelines for submitting code & software for further information.

Data

Policy information about availability of data

All manuscripts must include a data availability statement. This statement should provide the following information, where applicable:

- Accession codes, unique identifiers, or web links for publicly available datasets
- A list of figures that have associated raw data
- A description of any restrictions on data availability

Expression microarray data are available at the Gene Expression Omnibus repository under accession number GSE106175 (https://www.ncbi.nlm.nih.gov/gds/?term=GSE106175). The data that support the findings of this study are available from the corresponding author, Alessandra d’Azzo, sandra.dazzo@stjude.org, upon request.

Field-specific reporting

Please select the one below that is the best fit for your research. If you are not sure, read the appropriate sections before making your selection.

☒ Life sciences ☐ Behavioural & social sciences ☐ Ecological, evolutionary & environmental sciences

For a reference copy of the document with all sections, see nature.com/documents/nr-reporting-summary-flat.pdf

Life sciences study design

All studies must disclose on these points even when the disclosure is negative.

Sample size	Sample sizes were used based on previous experience and standards within the field to have sufficient number of samples to be able to reach statistical significance.
Data exclusions	No data were excluded.
Replication	All the in vitro experiments were replicated with cells at different passage number and in different days.
Randomization	Cells were randomly selected to be treated with DMSO or with SAHA or romidepsin.
Blinding	For the score of immunohistochemistry stainings, the pathologist was blinded to the genotype group.

Reporting for specific materials, systems and methods

We require information from authors about some types of materials, experimental systems and methods used in many studies. Here, indicate whether each material, system or method listed is relevant to your study. If you are not sure if a list item applies to your research, read the appropriate section before selecting a response.

Materials & experimental systems

n/a	Involved in the study	
☐	☒	Antibodies
☐	☒	Eukaryotic cell lines
☒	☐	Palaeontology
☒	☐	Animals and other organisms
☒	☐	Human research participants
☒	☐	Clinical data

Methods

n/a	Involved in the study	
☒	☐	ChIP-seq
☐	☒	Flow cytometry
☒	☐	MRI-based neuroimaging

Antibodies

Antibodies used	anti-MYC (Cell Signaling 9402), anti-Lamp1 (BD 553792), anti-LAMP1 (Cell Signaling 9091), anti-LC3B (Cell Signaling 3868), anti-Flag M2 (Sigma F1804), anti-HDAC2 (Abcam 7029, Cell Signaling 2545), anti-TFEB (Bethyl laboratories Inc A303-672A), anti-TFE3 (Sigma-Aldrich HPA023881), anti-NEU1 and anti-PPCA antibodies were generated in-house. anti-Oct4 (Santa Cruz Biotechnologies, sc-5279, clone No. C-10), anti-Nanog (Santa Cruz Biotechnologies, sc-33759), anti-SSEA4 (Millipore, MAB4304), anti-SSEA3 (Millipore, MAB4303-I), anti-TRA-1-60 (Millipore, MAB4360)
Validation	Validation of the above mentioned antibodies is available on the manufacturer's website; for immunohistochemistry or immunofluorescence analyses validation has been done by incubating samples with either the primary Ab or blocking buffer followed by detection with proper secondary antibody.

Eukaryotic cell lines

Policy information about cell lines

Cell line source(s)	Human cell lines HeLa and RH30 (available at ATCC) were provided by Dr. Grosveld (St. Jude Children's Research Hospital), Sy5y, available at ATCC, was provided by Dr Freeman (UTHSC), control fibroblasts were purchased from Coriell Institute. Human sialidosis fibroblasts received by my laboratory were uncoded and unidentifiable. They came from the Rotterdam Biobank (Rotterdam, The Netherlands), the Pediatric Undiagnosed Diseases Program, National Human Genome Research Institute/NIH (Bethesda MD, USA) and the Muscle Unit Section and Laboratory of Skeletal Muscle Pathology Department of Neurology, Medical School of the University of São Paulo (São Paulo, Brazil).
Authentication	Not authenticated

Mycoplasma contamination	All cell lines were micoplasma free
Commonly misidentified lines (See ICLAC register)	No commonly misidentified lines were used in this study

Flow Cytometry

Plots

Confirm that:

☒ The axis labels state the marker and fluorochrome used (e.g. CD4-FITC).

☒ The axis scales are clearly visible. Include numbers along axes only for bottom left plot of group (a 'group' is an analysis of identical markers).

☒ All plots are contour plots with outliers or pseudocolor plots.

☒ A numerical value for number of cells or percentage (with statistics) is provided.

Methodology

Sample preparation	For FACS analysis , cultured cells were collected after trypsinisation and processed as described in the Methods section.<br>For flow cytometry, cells were stained following protocols described in the Methods section.
Instrument	FACS: BD Biosciences Aria cell sorter<br>Flow cytometry: BD Biosciences LSRFortessa intrument
Software	FACS data were collected using Diva software version 8.0.1. FlowJo version 10.57 was used for data analysis.
Cell population abundance	Abundance and purity of the relevant cell population were assessed by flow cytometry analysis. Cells with purity > 85% were used.
Gating strategy	Negative controls (isotype match antibodies, Lysotracker negative or EGFP-negative cells) were used to determine negative and positive boundaries. Cells were first gated for live cells using DAPI vs. FSC-A. Live cells were then gated using a single cell gate using FSC-A vs. SSC-W. Lastly, single, live cells were analyzed for fluorescent intensity using a histogram plot with the marker of interest (i.e. Lysotracker green or EGFP) on the x-axis and a gate marking the positive and negative boundaries.

☐ Tick this box to confirm that a figure exemplifying the gating strategy is provided in the Supplementary Information.
